# Induction of Bioactive Secondary Metabolites in *Annona cherimola* Mill. (Chirimoya) Seedlings Using Chitosan and Salicylic Acid as Elicitors

**DOI:** 10.1002/pei3.70075

**Published:** 2025-08-18

**Authors:** Mónica F. Antolínez‐Pérez, Juan P. Fernández‐Trujillo, Luis G. Sequeda‐Castañeda, Yineth Pineros‐Castro

**Affiliations:** ^1^ Natural Sciences and Engineering Faculty Universidad Jorge Tadeo Lozano Bogotá Colombia; ^2^ Department of Agronomical Engineering Universidad Politécnica de Cartagena Cartagena España; ^3^ Department of Chemistry, Faculty of Sciences Pontificia Universidad Javeriana Bogotá Colombia

**Keywords:** *Annona cherimola*, antioxidant activity, bioactive compounds, biotic stress, chitosan, salicylic acid

## Abstract

The use of elicitors such as salicylic acid (SA) and chitosan (CH) represents an effective strategy to induce the production of secondary metabolites in plants. However, its application in seedlings of *Annona cherimola* Mill. has been scarcely explored. This study evaluated the effect of different concentrations of SA (100, 250 and 400 μM) and CH (60, 80 and 100 μM) applied foliar on phenological growth, accumulation of phenolic compounds and flavonoids, and antioxidant activity in *Annona cherimola* . Three weekly treatments were applied under controlled conditions. Total phenolic content (TPC) was quantified as milligrams of gallic acid equivalents per gram of dry mass (mg GAE/g DW) of extract, and total flavonoid content (TFC) as milligrams of quercetin equivalents per gram of dry mass (mg QE/g DW) of extract. Antioxidant activity was determined using the 2,2‐diphenyl‐1‐picrylhydrazyl (DPPH) radical scavenging method and the ferric reducing antioxidant power (FRAP) assay, and results were expressed as milligrams of Trolox equivalents per gram of dry mass (mg TE/g DW) of extract. The results showed that salicylic acid 100 μM and chitosan 80 μM promoted the highest stem growth and leaf count. These concentrations also induced the highest content of phenols (up to 23.5 mg GAE/g DW) and flavonoids (15.5 mg QE/g DW), as well as higher antioxidant activity (up to 21.5 mg TE/g DW). A positive correlation was observed between the accumulation of metabolites and the antioxidant capacity. This is the first report to validate the use of salicylic acid and chitosan as biostimulant tools to modulate physiology and secondary metabolism in *Annona cherimola* (Chirimoya).

## 1 Introduction

*Annona cherimola* Mill., known as cherimoya, is a species belonging to the Annonaceae family, widely recognized for its nutritional, ethnomedicinal, and pharmacological properties (Amoo et al. 2008; González‐Vega 2013; Jamkhande et al. 2017; Perrone et al. 2022; Yassin et al. 2025). Although its cultivation is distributed in subtropical regions of America, Asia, and Europe, its industrial use has been mainly limited to the fruit, despite the fact that various parts of the plant, such as leaves and seeds, contain bioactive compounds of interest such as acetogenins, flavonoids, terpenoids, amino acids, organic acids, carbohydrates, cholines, acids, phenylpropanoids, and alkaloids, among others (Albuquerque et al. 2016; Díaz‐de‐Cerio et al. 2018; Yajid et al. 2018; Badmus et al. 2020; Aguilar‐Villalva et al. 2021; Humbal and Pathak 2023). These secondary metabolites have demonstrated antioxidant, antimicrobial, cytotoxic, immunosuppressive, insecticide, antidiabetic, antiulcer, and anthelmintic potential and in the treatment of neurological disorders, which highlights the need to explore strategies to increase their production in the early stages of plant development (Radman et al. 2003; Barboza et al. 2014; Perrone et al. 2022; Zubaidi et al. 2023; Dey et al. 2024).

Synthesis of secondary metabolites is closely related to adaptive responses to environmental stress. In this context, the use of elicitors—organic and inorganic substances capable of inducing physiological and biochemical responses in plants—has emerged as an effective biotechnological strategy to stimulate the accumulation of functional compounds (Radman et al. 2003; Zhao et al. 2005; Garcia‐Brugger et al. 2006; Henry et al. 2012; Barboza et al. 2014; Kandoudi and Németh‐Zámboriné 2022). The application of elicitors in plants generates a signal at the level of the cell membrane, activates different secondary metabolic pathways, and produces a series of biochemical activities such as the generation of reactive oxygen species (ROS), protein accumulation, cell wall (structural changes), hypersensitivity reaction (cell death at the site of infection), activation of defense response genes, synthesis of defense molecules (tannins and phytoalexins), synthesis of secondary messengers (jasmonic acid and salicylic acid), and acquired systemic resistance (SAR) which makes them more resistant to subsequent stress‐inducing attacks (Walters et al. 2013; Wiesel et al. 2014; Reshi et al. 2023).

Salicylic acid (SA), a plant hormone that acts as an endogenous signal in defense against pathogens, and chitosan (CH), a chitin‐derived polysaccharide with biostimulant capacity, have been used in various species to improve physiological and biochemical performance, promoting the synthesis of phenols, flavonoids, and other defense molecules (Khurizadeh et al. 2024; Linares‐Castañeda et al. 2025; Tian et al. 2025; Zhang et al. 2025).

Recent studies have shown that the addition of SA to culture media can improve the production of phenolic compounds, increase antioxidant activity, and activate enzyme defense systems. For instance, *in Arnica montana* in vitro shoots, Petrova et al. (2025) reported that supplementation with 100 μM SA induced vigorous shoot growth, although it downregulated caffeoylquinic acid and total phenolic levels (Petrova et al. 2025). In *Pelargonium graveolens* callus cultures, Elbouzidi et al. (2025) found that treatment with 25 μM SA significantly enhanced total phenolic and flavonoid content, as well as antioxidant activities (Elbouzidi et al. 2025). At the agronomic level, exogenous application of SA at 200 ppm (approximately 1.45 mM) has also been validated as a disease management strategy, reducing the incidence of late wilt of corn caused by *Magnaporthiopsis maydis* and increasing crop yield under field conditions (Elshahawy and Abd El‐Wahed 2025).

Biotic elicitors such as CH have gained relevance due to their low cost, biocompatibility, and ability to induce defense responses in plants (Stasińska‐Jakubas and Hawrylak‐Nowak 2022). Several recent studies have documented their efficacy in species of medicinal or ornamental interest. For example, Monica and Kumaria (2025) reported a significant increase in germination, shoot formation, and roots in *Cymbidium aloifolium* by supplementing the culture medium with 3–5 mg/L of CH, while maintaining the genetic fidelity of the regenerated seedlings (Monica and Kumaria 2025). In another approach, Javed et al. (2025) showed that CH (5 mg/L) promotes the accumulation of phenols, flavonoids, and alkaloids in *Rhazya stricta calluses*, with antioxidant and cytotoxic effects relevant to H1299 lung cancer cell lines (Javed et al. 2025). For their part, Mueangnak et al. (2025) identified that both CH (5 mg/L) and CuSO₄ (6.4 μM) stimulate the production of betalains with high antioxidant capacity in cultures suspended with *Celosia argentea* (Mueangnak et al. 2025).

Based on the available literature, studies on the application of elicitors in seedlings of *Annona cherimola* are limited or have not yet been reported (Perrone et al. 2022). Despite the recognition of its leaves as a source of bioactive metabolites, the response of this species to treatment with foliar elicitors under in vivo conditions, especially during the vegetative phase, has not yet been fully characterized (Mannino et al. 2020). The optimal concentration of these compounds to induce the accumulation of antioxidants in this species is also not precisely known. From a biotechnological perspective, it is essential to develop protocols that allow the production of secondary metabolites in seedlings to be induced in a controlled manner, with a view to their potential use in the formulation of natural products. This study hypothesizes that the application of elicitors such as SA and CH stimulates the biosynthesis of secondary metabolites in the leaves of *Annona cherimola* seedlings, reaching levels comparable to those observed in mature trees. To test this hypothesis, the effects of different concentrations of SA and chitosan CH on phenological growth, phenol and flavonoid content, and antioxidant activity were evaluated in *Annona cherimola* seedlings.

## 2 Materials and Methods

During the conduct of the experiments, the safety and hygiene standards established by national legislation and institutional guidelines were strictly followed. Likewise, preventive measures were implemented to minimize the risks associated with the handling of the materials and reagents used.

### 2.1 Reagents and Standards

The following Sigma‐Aldrich reagents and standards were used: Salicylic acid (≥ 99.0%, product number 247588), Chitosan (≥ 99.9%, product number 419419), Gallic acid (≥ 99.8%, product number G7384), Quercetin (≥ 95.8%, product number Q4951), (±)‐6‐Hydroxy‐2,5,7,8‐tetramethylchromane‐2‐carboxylic acid (Trolox, ≥ 97.8%, product number 238813), 2,2′‐Azino‐bis(3‐ethylbenzothiazoline‐6‐sulfonic acid) diammonium salt (DPPH, ≥ 98.5%, product number A1888), 2,4,6‐Tris(2‐pyridyl)‐s‐triazine (TPTZ, ≥ 98.0%, product number 93285), Folin‐Ciocalteu reagent (2.0 N, product number F9252), and Tween^R^ 80 (product number P4780).

### 2.2 Experimental Design and General Conditions

A completely randomized experiment was designed with seven treatments (three concentrations of CH, three concentrations of SA and one control) and seven replicates per treatment (*n* = 7). The experimental units corresponded to individual seedlings of *Annona cherimola* Mill. of 15 months of age, acquired in the local market of Tunja (Boyacá, Colombia) and kept in a greenhouse under controlled conditions (25°C ± 2°C, 70% RH, photoperiod 16: 8 h light/dark).

### 2.3 Application of Treatments

The following concentrations were evaluated: SA at 100, 250, and 400 μM, and CH at 60, 80, and 100 μM, dissolved in distilled water with 1 mL·L^−1^ of Tween 80 as surfactant. CH was solubilized in water acidified (0.1% acetic acid). The concentrations used were chosen based on previous studies (Khan et al. 2002; Jayaraj et al. 2009). 50 mL of elicitor solution per plant was applied by foliar spraying, making three applications (day 0, 7 and 14). The control group was treated only with distilled water and Tween 80 in the same proportion.

### 2.4 Evaluation of Phenological Indicators

Two response variables were monitored: total number of leaves and stem elongation (from base to apex) on days 0, 7, 14, and 21. Data were expressed as a percentage change from baseline: Δ% = [(Value on Day X—Baseline (Day 0)) / Baseline (Day 0)] × 100.

### 2.5 Harvesting, Drying, and Extraction of Plants

At 21 days after the start of the experiment, six branches were randomly collected per plant, including primary, secondary, tertiary, and terminal leaflets. The plant material was dried at 30°C for 96 h, ground (0.75 mm), and stored in dark bags at 4°C. 0.2 g of each sample was taken and extracted with 3 mL of 96% ethanol for 96 h at room temperature in dark vials with daily manual stirring (Gleye et al. 1997; ElNaker et al. 2021; Naviglio et al. 2023).

### 2.6 Quantification of Secondary Metabolites and Antioxidant Activity

Total phenol analysis was performed using the Folin‐Ciocalteu method, determining the absorbance at 765 nm (Singleton et al. 1999). Results are expressed in milligrams of gallic acid equivalent per gram of dry mass (mg GAE/g DW). The determination of flavonoids was performed using the aluminum chloride method, in which the absorbance was determined at 415 nm (Mammen and Daniel 2012; Cacique et al. 2021). The results are expressed in milligrams of quercetin equivalent per gram of dry mass (mg QE/g DW) of extract.

Antioxidant activity by the DPPH (2,2‐diphenyl‐1‐picrylhydrazil) method was determined following the methodology proposed by Brand‐Williams et al. (1995), which is based on the reduction of absorbance measured at 517 nm. Results are expressed in milligrams of Trolox equivalent per gram of dry mass (mg TE/g DW) of extract (Brand‐Williams et al. 1995; Cacique et al. 2021). For antioxidant activity using the FRAP (Ferric Reducing Antioxidant Power) method, it was determined according to the methodology proposed by Benzie and Strain (1996). Results are expressed in milligrams of Trolox equivalent per gram of dry mass (mg TE/g DW) of extract (Benzie and Strain 1996; Cacique et al. 2021).

### 2.7 Statistical Analysis

Data were processed using one‐way ANOVA and polynomial regression analysis (linear, quadratic, and cubic effects) using Design‐Expert 13.0, SPSS Statistics, and Minitab 20 programs. When relevant, multiple comparisons with Tukey's test or LSD, with a significance level of *p* < 0.05, were conducted. Significant effects were reported according to the trend (L, Q, C) and the number of replicates per group was included (Little 1981; IBM‐Corp 2022).

## 3 Results

### 3.1 Effect of Elicitors on Phenological Indicators

**FIGURE 1:**
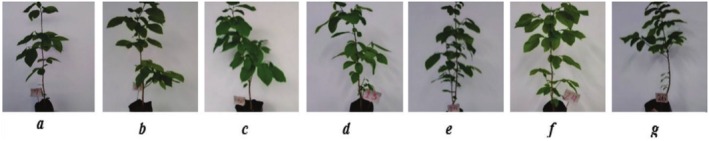
Representative seedlings of each treatment group exposed to salicylic acid (SA) and chitosan (CH) at different concentrations (21 days of experiment): (a) Control; (b) SA 100 μM; (c) SA 250 μM; (d) SA 400 μM; (e) CH 60 μM; (f) CH 80 μM; (g) CH 100 μM.

**FIGURE 2:**
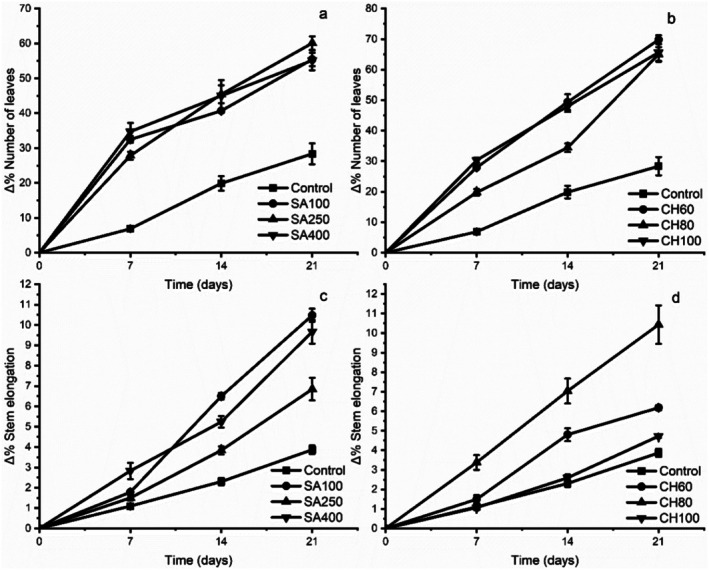
Effect of the elicitors salicylic acid (SA) and chitosan (CH) on the number of leaves (a, b) and stem elongation (c, d), expressed as a percentage of increase relative to the initial values during the experimental period. SA and CH concentrations (μM).

Treatments with SA and CH significantly modified *Annona cherimola* seedlings, both in stem elongation and in the number of leaves (*p* < 0.05) (Figure 1). Significant differences were observed between treatments and evaluation times, as well as in the interaction concentration × time (Figures 1 and 2).

The Δ% shown in Figure 2 refers to the percentage of relative increase with respect to the initial value (day 0) for each variable (number of leaves or elongation of the stem). Figure 2 shows the effect of SA and CH on leaf number (panels a and b) and stem elongation (panels c and d) in *Annona cherimola* Mill. over 21 days, expressed as a percentage increase relative to the initial values.

Regarding the number of leaves, SA treatments (Figure 2a) showed a progressive increase over time. Treatment with SA 100 promoted the greatest increase, reaching 60% on day 21, followed by SA 250 (56%) and SA 400 (29%), while the control group recorded an increase of 35%. No marked differences were observed between SA 100 and SA 250, but there was an evident decrease with SA 400, suggesting a negative effect at high concentrations. For CH (Figure 2b), CH 80 was the treatment that promoted the highest number of leaves with a 69% increase, followed by CH 60 (67%) and CH 100 (63%), while the control group showed the lowest value (28%). This indicates that all the evaluated CH concentrations significantly stimulated leaf production, with CH 80 being the most effective.

Regarding stem elongation, SA treatments (Figure 2c) showed a pattern similar to that observed for the number of leaves. SA 100 promoted the largest increase (10.4%), followed by SA 250 (9.8%) and SA 400 (6.8%), compared to the control (4.3%). In the case of CH (Figure 2d), CH 80 induced the highest stem elongation (11.2%), outperforming CH 60 (8.3%), CH 100 (6.1%) and the control (3.7%). This response suggests that the intermediate concentration of chitosan is the most efficient in promoting height growth.

In summary, both SA and CH promoted leaf formation and stem elongation in *Annona cherimola* seedlings, being more effective at intermediate concentrations (SA 100 and CH 80), possibly due to the activation of hormonal pathways associated with auxins and cytokinins, in synergy with the effects of induced defense (Naseem et al. 2015; Hurný et al. 2020). Higher concentrations of both elicitors showed lower efficacy, which could be associated with phytotoxic effects or physiological overstimulation (Golkar et al. 2019; Elbouzidi et al. 2025).

### 3.2 Effect of Elicitors on Secondary Metabolite Production and Antioxidant Activity

**FIGURE 3:**
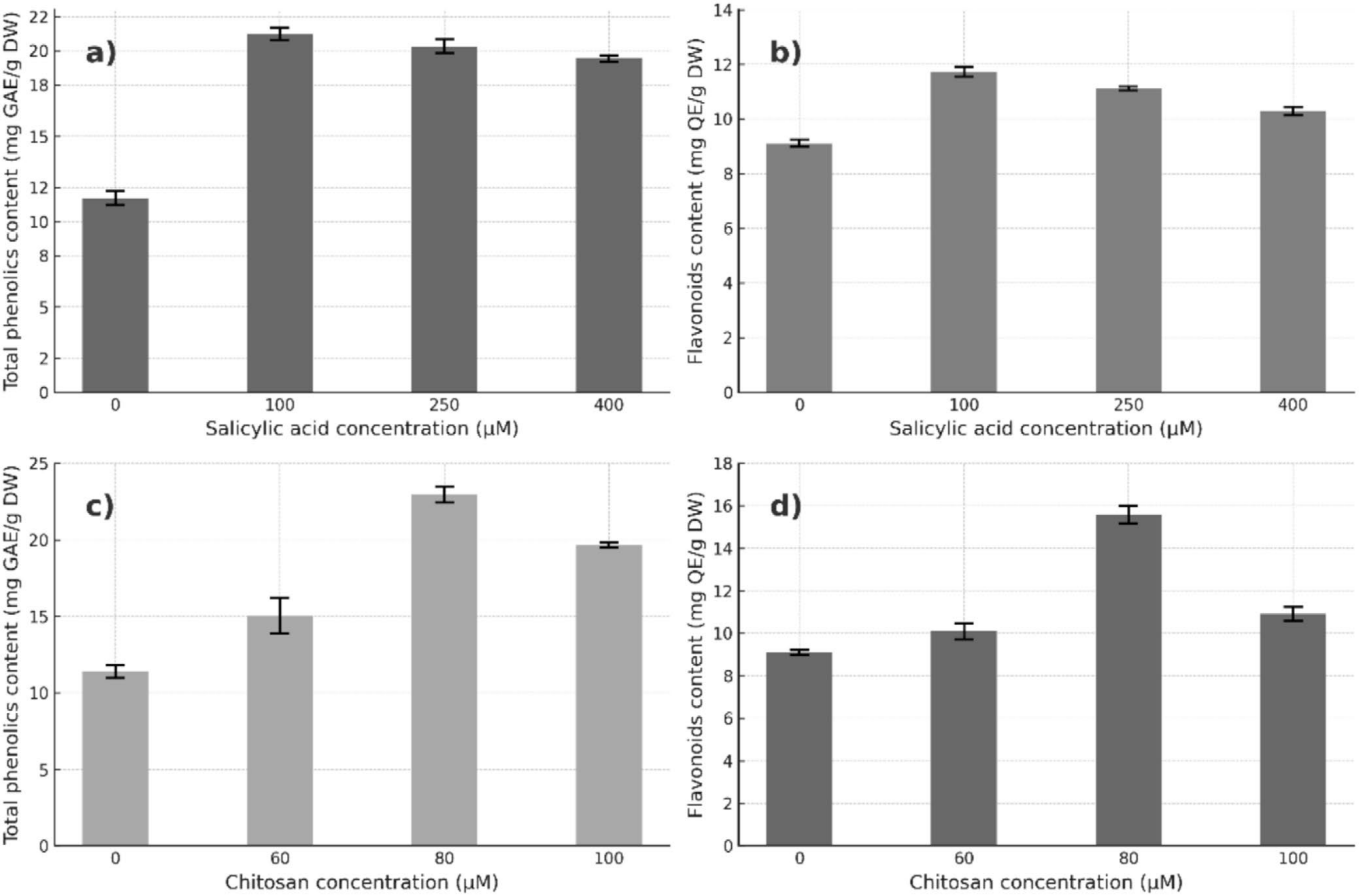
Effects of the elicitors salicylic acid (SA) and chitosan (CH) on total phenolic content (a–c) and total flavonoid content (b–d) in the leaves of *Annona cherimola* Mill. Data are expressed as mean ± standard deviation (*n* = 7). All response patterns followed significant linear, quadratic, and cubic trends.

Treatments with SA and CH promoted a significant increase (*p* < 0.05) in the contents of phenolic compounds and flavonoids in the treated seedlings (Figure 3). The observed trends depended on the concentration of the elicitor, following significant polynomial models (L, Q, C) for both compounds (*p* < 0.05).

In the case of SA, the concentration of 100 μM induced the highest content of total phenols, reaching approximately 22.5 mg GAE/g DW, which represents an increase of 114.3% compared to the control group (10.5 mg GAE/g DW). Similarly, the total flavonoid content increased to 12.3 mg QE/g DW at the same concentration, with an increase of 39.8% compared to the control (8.8 mg QE/g DW).

However, treatment with CH at 80 μM produced the highest total phenol content (~23.5 mg GAE/g DW), with an increase of 80.8% compared to the control (13.0 mg GAE/g DW). Regarding flavonoids, a maximum value of 15.5 mg QE/g DW was observed, which corresponds to an increase of 72.2% compared to the baseline value of 9.0 mg QE/g DW. These results show a dose‐dependent response of phenolic metabolites to the application of elicitors, suggesting an active role for SA and CH in the activation of secondary metabolism related to antioxidant defense mechanisms.

**FIGURE 4:**
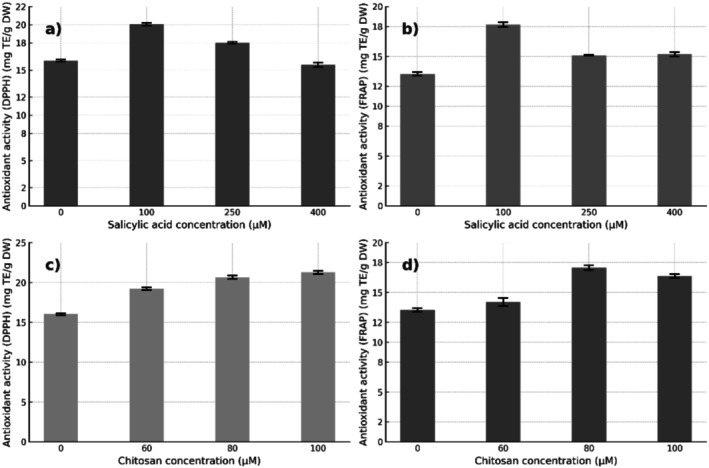
Antioxidant activity (AA) in leaves of *Annona cherimola* Mill. treated with salicylic acid (SA) and chitosan (CH). The free radical scavenging activity was evaluated using the DPPH assay (a–c) and the FRAP assay (b, d). Data are expressed as mean ± standard deviation (*n* = 7). Polynomial regression analysis revealed significant linear, quadratic, and cubic trends (*p* < 0.05) for both DPPH and FRAP, except for the quadratic term of DPPH in leaves treated with chitosan.

Regarding the antioxidant activity determined by the DPPH and FRAP methods, significant increases were observed in response to the SA and CH treatments (Figure 4). In the DPPH trial, SA treatment at 100 μM promoted the highest antioxidant activity, with an approximate value of 21.5 mg TE/g DW, which represents an increase of 59.3% compared to the control (13.5 mg TE/g DW). Similarly, in the FRAP trial, the highest activity was also recorded with 100 μM SA, reaching approximately 18.5 mg TE/g DW, with an increase of 42.3% compared to the control group (13.0 mg TE/g DW). On the other hand, chitosan at 100 μM induced a comparable effect on DPPH activity (21.5 mg TE/g DW), with an increase of 59.3% compared to the control. In the FRAP trial, the greatest effect was observed with 80 μM CH, with a value of 18.5 mg TE/g DW and a 37.0% increase over the control (13.5 mg TE/g DW). These results show that both elicitors significantly improved the antioxidant capacity of the seedlings, with DPPH being more sensitive to detect such changes.

A positive correlation was evidenced between the content of total phenols, total flavonoids, and the antioxidant activity determined by the DPPH and FRAP methods. The concentrations of salicylic acid and chitosan that promoted the highest levels of phenolic compounds (100 and 80 μM, respectively) coincided with the highest values of antioxidant activity.

The results suggest that both elicitor‐induced phenols and flavonoids are closely related to the improvement of antioxidant capacity, which is consistent with their known role as free radical scavenging agents and electron donors (Golkar et al. 2019; Elbouzidi et al. 2025).

## 4 Discussion

The results obtained in this study show that the foliar application of SA and CH in *Annona cherimola* significantly promotes both vegetative growth and the accumulation of secondary metabolites with antioxidant activity. These physiological and biochemical responses allow us to infer that both elicitors stimulate defense mechanisms mediated by induced plant signaling pathways (Murthy et al. 2014; Akif Açıkgöz 2025; Mubeen et al. 2025).

From a morphological point of view, stem elongation and leaf number increased with SA (100 μM) and CH (80 μM) treatments, with significant differences compared to the control. This behavior coincides with previous studies that describe the role of AS as a modulator of growth and acquired systemic resistance in various species (Rivas‐San Vicente and Plasencia 2011; Yusuf et al. 2013; Dempsey and Klessig 2017; Rani and Murali‐Baskaran 2025), as well as research that attributed to CH a biostimulant effect on photosynthetic rate and plant morphogenesis (El Hadrami et al. 2010; Yin et al. 2012; Nguyen Van et al. 2013; Rani and Murali‐Baskaran 2025). The magnitude of the observed effect suggests that young *Annona cherimola* seedlings respond favorably to low concentrations of these elicitors, which is relevant for the initial stages of propagation in production systems (Murthy et al. 2014; López‐Peñuelas et al. 2025).

SA has been documented to increase growth in several species: 150% in *Capsicum annuum* (pepper) (Chandrasekhar et al. 2017), 14.8% in *Lycopersicon esculentum* Mill. (tomato) (Larqué‐Saavedra et al. 2010), and 6% in *Cicer arietinum* L. (chickpea) (Saikia et al. 2003). Significant increases in leaf number have also been observed in *Saintpaulia ionantha* Wendl. (African violet) (Martín‐Mex et al. 2005) and *Fragaria* × *ananassa* (strawberry) (Anchondo‐Aguilar et al. 2011). CH‐induced stimulation of vegetative growth in our seedlings is consistent with the findings in *Origanum vulgare* ssp. *hirtum* (Greek oregano) (Yin et al. 2012), *Fragaria* × *ananassa* (strawberry) (El‐Miniawy et al. 2013) and *Coffea canephora* var. *robust* (Nguyen Van et al. 2013). In the latter, 10 ppm of CH nanoparticles increased 1.11 times the height of the plant and 1.02 times the diameter of the stem compared to the control. In addition, the addition of CH of different molecular weights to the soil increased the height, diameter, and leaf area in the soil. *Capsicum annuum* L. (Chookhongkha et al. 2012).

Regarding the phytochemical profile, a significant increase in the contents of phenolic compounds and flavonoids was observed, especially with chitosan treatment at 80 μM. These metabolites are closely related to plant defense mechanisms against biotic stress, acting as antioxidants, hormone regulators, and signaling molecules (Karabourniotis et al. 2014; Golkar et al. 2019; Rani and Murali‐Baskaran 2025). The levels detected in this study (23.5 mg GAE/g DW) were higher than those reported by Mannino et al. (2020) in adult leaves of *Annona cherimola* , where total phenolic contents ranged from approximately 5.7–8.4 mg GAE/g DW, depending on the cultivar analyzed (Mannino et al. 2020). These differences may be attributed to factors such as phenological stage, growth microclimate, extraction methodology, and experimental conditions (Ghasemzadeh et al. 2016).

SA and CH play a key role in the induction of polyphenols. SA promotes the accumulation of secondary metabolites through gene regulation and endogenous signaling (Fazal et al. 2016; Ali 2021), which has been documented in species such as *Coleus aromaticus* (Govindaraju and Indra Arulselvi 2018), *Achillea millefolium* (Gorni and Pacheco 2016) and *Mentha piperita* (Figueroa‐Pérez et al. 2014). Our results are consistent with these studies, although CH generated higher levels of metabolites.

The application of CH has been shown to activate genes related to polyphenol synthesis, with considerable increases observed in cell cultures and in vitro (Hidangmayum et al. 2019; Khan et al. 2019). However, there are few in vivo studies. In *Origanum vulgare* ssp. *hirtum* (Greek oregano) foliar application of 50 ppm CH increased phenols by 38% and flavonoids by 29% (Yin et al. 2012), while in *Ocimum basilicum* L. (basil) CH favored the accumulation of phenols and terpenoids by 2.5 times (Kim et al. 2005). These effects are attributed to the regulation of key enzymes in phenolic biosynthesis (Ferri et al. 2009).

CH application has been shown to activate key biosynthetic pathways leading to the accumulation of phenols and flavonoids in various plant systems. Among the most important enzymes induced is phenylalanine ammonium lyase (PAL), which catalyzes the initial step of the phenylpropanoid pathway converting phenylalanine to cinnamic acid, and whose activity was increased up to 32‐fold in *Ocimum basilicum* after CH treatment (Kim et al. 2005). Likewise, chalcone synthase (CHS) and chalcone‐flavanone isomerase (CHI) have been identified as key enzymes in the derivatization of metabolites towards flavonoid biosynthesis, showing a direct correlation between their expression and the accumulation of anthocyanins and other flavonoids (Ferri et al. 2009). Furthermore, in *Vitis vinifera* , CH stimulates the activity of stilbene synthase (STS), responsible for the synthesis of stilbenes such as resveratrol (Ferri et al. 2009). Complementarily, although not directly involved in phenolic biosynthesis, the antioxidant enzymes peroxidase (POD) and superoxide dismutase (SOD) are frequently induced following CH application, reflecting a generalized enhancement of defense responses associated with phenolic metabolism (Khan et al. 2019). Taken together, these results provide evidence that CH acts as a potent elicitor of the phenylpropanoid pathway, increasing the activity of key biosynthetic enzymes and antioxidants, and thus favoring the accumulation of bioactive phenolic compounds in plants (Yin et al. 2012; Hidangmayum et al. 2019).

CH showed greater efficacy in the accumulation of antioxidant compounds than SA, which could be due to its ability to induce additional biosynthetic pathways related to the production of phytoalexins, pathogen‐related proteins, and cellular lignification (Ferri et al. 2009; Hidangmayum et al. 2019). This finding suggests that CH acts as a more potent elicitor under the conditions evaluated, although the response could also be modulated by the stage of seedling development, exposure time, and cumulative dosage (Venkatasai et al. 2025).

In relation to antioxidant activity, the values recorded in this study were lower than those reported for adult leaves of *Annona cherimola* , where Mannino et al. (2020) reported antioxidant activities that reached 39 mg TE/g DW, depending on the method used and the cultivar analyzed (Mannino et al. 2020). These differences are mainly due to the phenological stage of the plants, since in juvenile stages the antioxidant systems are still developing and have not reached the maximum capacity for synthesis and accumulation of secondary metabolites (Ghasemzadeh et al. 2016). This finding highlights the importance of considering the developmental stage when evaluating the antioxidant potential of species of agroindustrial and pharmaceutical interest, and reinforces the need to study secondary metabolism as a highly dynamic phenomenon, dependent on both plant development and environmental conditions (Rao and Zheng 2025). The observed correlation between increased phenolic metabolites (phenols and flavonoids) and antioxidant capacity (DPPH and FRAP) indicates a coordinated activation of secondary metabolism induced by treatments (Weng and Huang 2014; Guzmán‐Ortiz et al. 2017), as reported in species such as *Mentha piperita* (Figueroa‐Pérez et al. 2014), *Achillea millefolium* (Gorni and Pacheco 2016) and *Origanum vulgare* (Yin et al. 2012).

This is the first study to report on the joint effect of SA and CH in *Annona cherimola* seedlings, constituting a relevant advance for its application in plant biofactories. The seedlings used in the experiment were 15 months old, and the results showed that they were capable of producing levels of secondary metabolites comparable to those found in mature trees. This finding highlights the potential of young plants as an attractive and efficient source for the production of bioactive compounds derived from cherimoya.

However, there are some limitations, such as: (a) Individual metabolites were not characterized by chromatographic techniques (HPLC and NMR), which prevents knowing the qualitative composition. (b) The number of replicates and the experimental design must be expanded for validation in the field. (c) Possible unmeasured physiological effects, such as oxidative damage or gene regulation, were not evaluated.

Future research could focus on elucidating the molecular mechanisms activated by these elicitors, identifying key metabolites using advanced analyses (LC‐MS, NMR), and exploring their application under biotic stress conditions or in reproductive stages.

Overall, the evaluated results confirm that the elicitors induce relevant physiological and metabolic responses in *Annona cherimola* seedlings, with potential application in propagation programs, biomanufacturing, and development of functional ingredients for nutraceutical, pharmaceutical, and cosmeceutical products.

## 5 Conclusions

The results of this study demonstrate the foliar application of SA (100 μM) and CH (80 μM) in *Annona cherimola* Mill. seedlings induce a physiological and biochemical response characterized by a significant increase in vegetative growth, as well as in the accumulation of phenolic compounds and flavonoids with antioxidant activity. Concentrations of 100 μM for SA and 80 μM for CH were the most effective under the conditions evaluated. This integrated response confirms that elicitors activate defense mechanisms in early stages of plant development and contribute to the accumulation of secondary metabolites with antioxidant function. Consequently, the use of these compounds as tools to modulate the physiology of *Annona cherimola* seedlings under controlled conditions is validated.

## Conflicts of Interest

The authors declare no conflicts of interest.

## Supporting information


Data S1.

## Data Availability

The data that supports the findings of this study are available in the Data S1 of this article.
